# Mapping Immunodominant Antibody Epitopes of Abrin

**DOI:** 10.3390/antib9020011

**Published:** 2020-04-27

**Authors:** Ron Alcalay, Reut Falach, Yoav Gal, Anita Sapoznikov, Tamar Sabo, Chanoch Kronman, Ohad Mazor

**Affiliations:** 1 Department of Biochemistry and Molecular Genetics, Israel Institute for Biological Research, Ness-Ziona 76100, Israel; rona@iibr.gov.il (R.A.); reutf@iibr.gov.il (R.F.); yoavg@iibr.gov.il (Y.G.); anitas@iibr.gov.il (A.S.); tamars@iibr.gov.il (T.S.); chanochk@iibr.gov.il (C.K.); 2 Department of Infectious Diseases, Israel Institute for Biological Research, Ness-Ziona 76100, Israel

**Keywords:** abrin, toxin, polyclonal antibodies, epitope mapping

## Abstract

Abrin, a toxin isolated from the seeds of *Abrus precatorius* (jequirity pea) is considered a biological threat agent by the Center for Disease Control and Prevention. To date, there is no effective postexposure treatment for abrin poisoning, and efforts are being made to develop an efficient vaccine and measures for postexposure therapy. Epitope mapping is widely applied as an efficient tool for discovering the antigenic moieties of toxins, thus providing invaluable information needed for the development of vaccines and therapies. Aiming to identify the immunodominant epitopes of abrin, several neutralizing antiabrin polyclonal antibodies were screened using a set of 15-mer peptides spanning the amino acid sequence of either the A or B subunits of abrin. Analysis of the antibody-binding pattern revealed 11 linear epitopes for the A subunit and 14 epitopes for the B subunit that are located on the surface of the toxin and thus accessible for antibody interactions. Moreover, the spatial location of several of these epitopes suggests they may block the galactose-binding pockets or the catalytic domain, thus neutralizing the toxin. These findings provide useful information and suggest a possible strategy for the development and design of an improved abrin-based vaccine and therapeutic antibodies.

## 1. Introduction

Abrin, a toxin isolated from the seeds of *Abrus precatorius* (jequirity pea), belongs to the family of Type 2 ribosome inactivating glycoproteins (RIP) [1]. As such, abrin consists of two subunits, the enzymatic A-chain (ATA) that depurinates a specific adenine residue of the 28S ribosomal RNA of the 60S subunit, thereby arresting protein synthesis; and the B-chain (ATB), a lectin that binds galactose residues at the cell surface, thereby mediating toxin internalization into the cells [2,3]. Owing to its high toxicity, relative ease of purification, and accessibility, abrin is considered a biological threat agent by the Center for Disease Control and Prevention (CDC). Indeed, over the past decade, terrorist plots involving the use of abrin as a biological threat agent were uncovered prior to their execution [4].

To date, there is no effective postexposure treatment for abrin poisoning, and efforts are made to develop an efficient vaccine and measures for postexposure therapy [5]. Interestingly, abrin and ricin toxin share a marked homology in their sequence (42% for their A-chain and 59% for the B-chain) [6], and there is well-established body of knowledge on the immunodominant epitopes of ricin. However, there is no known cross-reactivity between antibodies elicited against ricin and abrin [7], and hence no shared neutralizing epitopes, reinforcing the need to map abrin epitopes. To date, only two neutralizing epitopes of antiabrin monoclonal antibodies were identified, both located on the surface of ATA [8,9].

Epitope mapping of the polyclonal antibodies in the sera of immunized animals is widely applied as an efficient tool for discovering antigenic moieties of pathogens, and thus provides invaluable information needed for the development of vaccines and therapies [10]. The aim of this work was to provide, for the first time, epitope-mapping analysis of several antiabrin polyclonal antibody preparations in order to identify the immunodominant epitopes on both subunits of the toxin.

## 2. Materials and Methods

### 2.1. Antibodies

Purified abrin was essentially prepared from *Abrus precatorius* seeds as described previously [9,11]. The immunization protocol of Serum R1 was described earlier [12], and that of Serum R3 (pooled from several immunized rabbits) was detailed by Sabo et al. [13]. Serum R2 was derived from a pool of rabbits that were immunized by three injections of alum-adsorbed abrin (4 µg per animal). M1 was derived from mice (CD-1 females) previously immunized by three injections of alum-adsorbed abrin (4 µg per animal), and the antibody-contained ascites fluid was collected and pooled.

### 2.2. ELISA Titer Determination

Determination of antiabrin antibody titers was performed as described before [12]. In short, maxisorp 96-well plates (Nunc, Sigma-Aldrich, St. Louis, MO, USA) were coated overnight with 2 µg/mL of abrin in 50 mM pH 9.6 carbonate–bicarbonate buffer, washed, and blocked with PBST buffer (0.05% Tween 20, 2% BSA in PBS). Antibodies were added and incubated in threefold dilutions for one hour; the plates were then washed with PBST and incubated with the reporting antibody (AP-conjugated-goat antirabbit or antimouse), and developed with substrate (p-nitrophenyl phosphate).

### 2.3. In Vitro Abrin-Neutralization Assay

Determination of antibody-neutralization potency was performed as described before [12]. In short, Ub-FL cells (a kind gift from Professor Piwnica-Worms University of Texas, MD Anderson Cancer Center, Austin, TX, USA) were seeded in 96-well plate (1.5 × 10^4^ cells/well) and incubated over night at 37 °C. Cell-culture medium was removed, and abrin (7 ng/mL) was added with serial dilutions of antiabrin antibodies. Twenty-four hours later, cell-culture medium was replenished with fresh medium containing proteasome inhibitor MG132 (Sigma, C2211 1 µM) for another hour. Cells were lysed by the addition of 50 µL lysis buffer (Promega, E1941), and residual luciferase activity was determined.

### 2.4. Epitope Mapping

A set of 15 amino acid long peptides (Figure S1), overlapping one another by 10 residues and spanning the sequence of either the A or the B subunits of abrin were produced by JPT Peptide Technologies (Berlin, Germany). Each peptide was biotinylated at the N-terminus and modified by glycine amide at the C-terminus. Lyophilized peptides were reconstituted using 100% DMSO and further diluted in PBST. Maxisorp 96-well microtiter plates were coated overnight with 5 µg/mL streptavidin, washed and blocked as described above. Peptides (5 µg/mL in PBST) were then added for 20 min, the plates were washed, and antiabrin antibodies diluted in PBST were added for 1 hour of incubation. Plates were then washed with PBST and incubated with the detecting antibody (AP conjugated goat antirabbit or antimouse) and developed with substrate (p-nitrophenyl phosphate).

## 3. Results

### 3.1. Characterization of Polyclonal Antiabrin Antibodies

As an initial step, we analyzed the binding properties of several polyclonal antiabrin-antibody preparations. These included ascitic fluid derived from mice immunized with purified abrin adsorbed on alum hydroxide (M1) and hyperimmune serum from rabbits immunized with abrin with Freund’s adjuvant (R1), abrin adsorbed on alum hydroxide (R2), or abrin adsorbed on alum hydroxide, followed by Freund’s incomplete adjuvant (R3).

To this end, binding of the different preparations to the toxin was assessed by ELISA, and the half-dilution values (Dil_50_) [13] were determined. Although all four preparations were found to bind abrin with high affinity (Dil_50_ of ~10,000 and above), the binding values of R1 and R3 were significantly higher than those of R2 and M1 (Table 1). Next, we in vitro determined the neutralizing potency of each preparation and assessed their ability to prevent abrin from arresting luciferase synthesis. Residual intracellular luciferase levels were measured, and the maximal dilution that allowed neutralization of 50% of abrin activity (ED_50_) was determined (Table 1). Overall, there was a positive correlation between the binding properties and the neutralization potencies of the tested preparations, where antibodies that exhibited high binding also possessed high PD_50_ values.

Interestingly, the proportion of the neutralizing antibodies in overall antiabrin antibodies (expressed as the ratio between binding and neutralization; B:N) in each preparation varied by up to sevenfold (1.4 to 9.4). Different vaccination strategies using the homologous toxin ricin elicited antibodies directed against the sugar moieties of the toxin to different degrees [14]. While antisugar antibodies increased the overall binding titer toward the toxin, they did not contribute to toxin neutralization. It may, therefore, follow that differences between B:N ratios reflect differences in the fraction of antisugar antibodies in various antiabrin preparations, an issue that we intend to assess in the future. Taken together, these results indicate that antiabrin preparations represent diverse sets of antibodies and are therefore suitable for fingerprinting the immunodominant epitopes of abrin.

### 3.2. Immunodominant Epitopes of Abrin Subunit A

**Figure 1:**
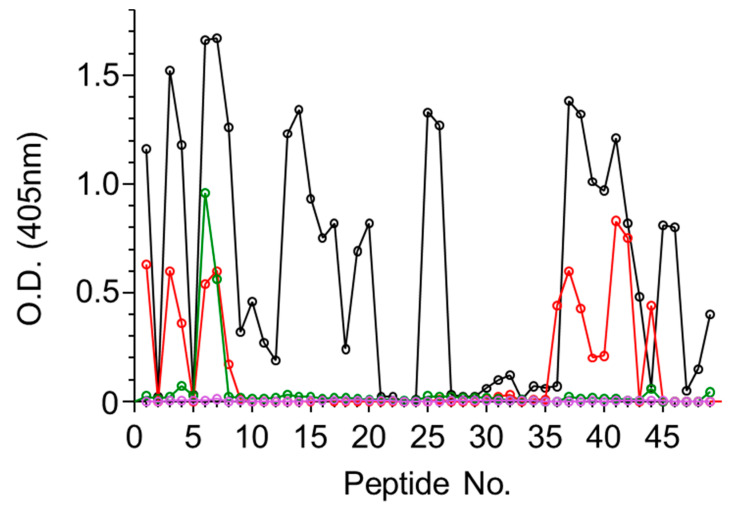
Binding of antiabrin sera to enzymatic A-chain (ATA) peptide array. Set of 15-mer biotinylated peptides spanning amino acid sequence of A subunit of abrin were immobilized on microtiter plates and incubated with antiabrin antibodies R1 (black), R2 (red), R3 (purple), or M1 (green). Plates were then washed, AP-conjugated secondary antibody was added, and antibody binding in each well was determined.

To characterize the polyclonal antibody response toward abrin, a set of 15-mer biotinylated peptides were prepared spanning the amino acid sequence of either the A or the B subunits of abrin, each peptide overlapping with the previous peptide by 10 amino acids, thus resulting in a set of 49 and 52 peptides for ATA and ATB, respectively (full sequences listed in Figure S1). The four antiabrin antibody preparations were first reacted with the ATA set of peptides and the binding to each peptide was determined. Since the peptides overlap each other, the epitope was considered positive only if it appeared in at least two successive peptides. The reactivity of Serum R1 toward ATA revealed the most diverse epitope recognition (Figure 1) that could be assigned to 11 sequences (Table 2).

**Figure 2:**
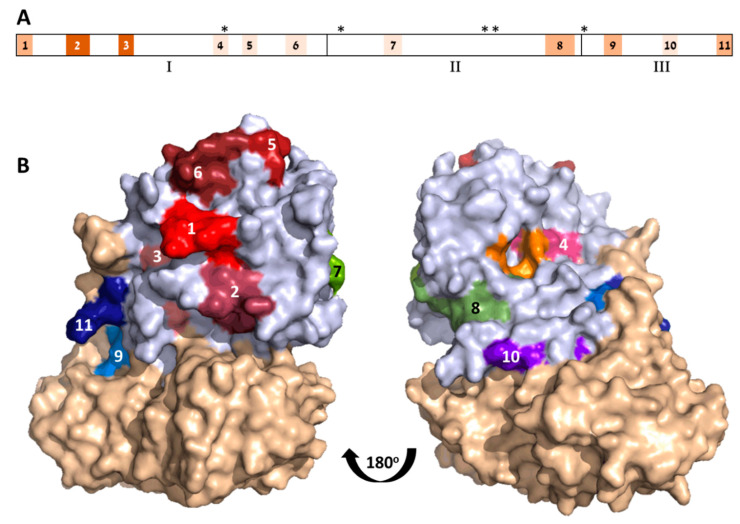
Immunodominant epitopes on ATA. (**A**) Linear depiction of ATA subunits folding domain (1–3) and amino acid residues (marked by asterisks) comprising catalytic domain. Location of immunodominant epitopes (1–11) marked as shaded boxes, whereas shading tones represent number of sera that reacted with each epitope (pale to darkest for 1 to 3 sera, respectively). (**B**) Crystal structure of abrin (PDB 1abr; ATA in pale blue and enzymatic B-chain (ATB) in light brown). Immunodominant epitopes color-coded and numbered; catalytic domain in orange.

The three-dimensional structure of ATA is classically divided into three folding domains: Domain 1 spans Residues 1–109, Domain 2 spans Residues 110–197, and Domain 3 spans Residues 198–251 [6]. According to this division, Domains 1–3 contain 6, 2, and 3 of the identified epitopes, respectively (Figure 2A).

While Serum R3 exhibited the highest titer and neutralization potency, it seems that it did not interact with any of the linear ATA epitopes (Figure 1). From the overall peptide-binding pattern, a response toward five epitopes could be deduced, all of which are shared with R1 (epitopes 1–3, 8, and 9). There was a significant response of Serum R2 with Peptide 44 that might suggest that there is another epitope located within that sequence. However, since this serum did not recognize the adjacent peptides that largely overlapped in the sequence, we could not relate the high response to a novel epitope. In contrast to these findings, the murine antiabrin antibodies reacted with only one major epitope (overlapping epitope 3) and Serum R3 did not react with any of the ATA peptides. These results may suggest that the ATA epitopes of these sera are mainly directed against nonlinear epitopes.

The location of the 11 ATA epitopes within the crystal structure of abrin is shown in Figure 2B. As expected from antibody epitopes, all 11 epitopes are located on the solvent-exposed surface of the toxin. In the majority of the cases, the exposed residues represent the full amino acid sequence of the predicted epitope. However, in some cases (i.e., Epitopes 3 and 10), only part of the assigned target epitope is located on the surface of the toxin, suggesting that, for these epitopes, amino acid residues that are in direct contact with the antibody are restricted, while other residues that are seemingly inaccessible are mainly responsible for maintaining the epitope 3D structure.

The toxicity of abrin stems from its catalytic activity that causes irreversible depurination of a specific adenine nucleotide within the 28S rRNA, thereby leading to the cessation of cellular-protein synthesis and eventually to cell death. This catalytic activity is mediated at the active site cleft within the A chain that consists of five residues (Y74, Y113, E164, R167, and W198) [6]. Though these residues map to noncontiguous sites within the linear sequence of abrin (Figure 2A), they cluster together to form the active site region (Figure 2B). It was, therefore, of interest to determine whether any of the mapped epitopes are located in the vicinity of the active site. Indeed, active-site Residue Y74 is part of Epitope 4, and that this epitope resides at the surface of the active site. It is thus tempting to assume that the antibody binding to this epitope blocks the active site, thereby directly neutralizing the catalytic activity of abrin. This notion may be supported by the study by Bagaria et al. [15] that mapped the epitope of an antiabrin monoclonal neutralizing antibody, D6F10. This antibody binds to Residues T112, G114, and R118 that are located also at the surface of the active site, contrapositioned to Epitope 4.

As mentioned earlier, very little is known about the targets of antiabrin-neutralizing antibodies; in fact, only two such epitopes, both located on ATA, have been described so far—epitopes of antibodies D6F10 (discussed above) and A7C4 ([9]. By using a set of toxin mutants, the authors concluded that Residues T82, G83, H85, D103, and H105 are crucial for the binding of this antibody. Here, we found that these residues are members of two of the identified immunodominant epitopes, Epitopes 5 and 6, respectively. Not surprisingly, in the folded form of the toxin, these two epitopes are adjacent to each other (Figure 2B), and they are positioned distal to the active site; however, to induce cell death, ATA needs to interact with other proteins en route to the cytoplasm (as was shown in detail for ricin subunit A [15]. It is, therefore, possible that binding antibodies to Epitope(s) 5 and/or 6 may interfere with one or more abrin:protein interactions required for ATA cytotoxic performance.

### 3.3. Immunodominant Epitopes of Abrin Subunit B

**Figure 3:**
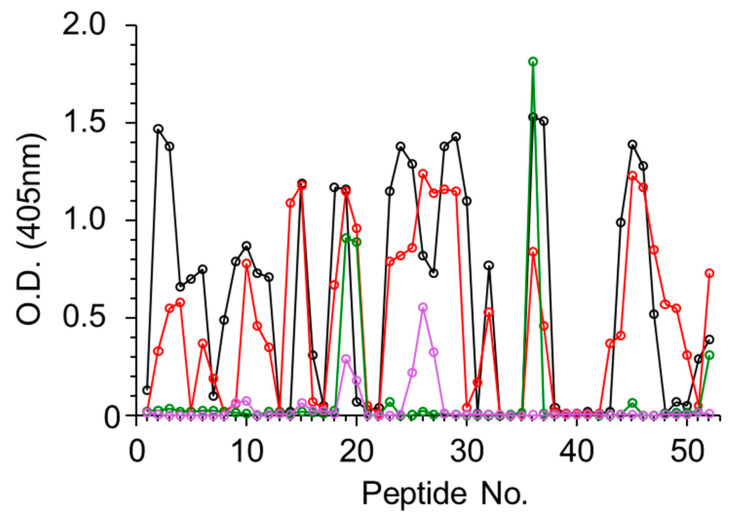
Binding of antiabrin sera to ATB peptide array. Set of 15-mer biotinylated peptides spanning amino acid sequence of B subunit of abrin were immobilized on microtiter plates and incubated with antiabrin antibodies R1 (black), R2 (red), R3 (purple), or M1 (green). Plates were then washed, AP-conjugated secondary antibody was added, and antibody binding in each well was determined.

Using the same strategy described above, the four antiabrin antibody preparations were allowed to interact with peptides spanning the amino acid sequence of the abrin B subunit (ATB). In this case, all four preparations interacted with the peptides (Figure 3), and 15 binding epitopes were identified overall (Table 3). Sera R1 and R3 exhibited diverse recognition with 13 shared epitopes (1–8, 10–13, and 15), whereas Serum R3 also interacted with Epitopes 9 and 14. Unlike the lack of interactions between Serum R2 and ATA, this serum was found to interact with two ATB epitopes, 7 and 9. The murine-derived antiabrin antibodies (M1) interacted with Epitopes 7, 12, and 15. The observation that all sera interacted with ATB epitopes, while only a limited number of these sera interacted with ATA epitopes, may imply that ATB is more immunogenic than ATA.

**Figure 4:**
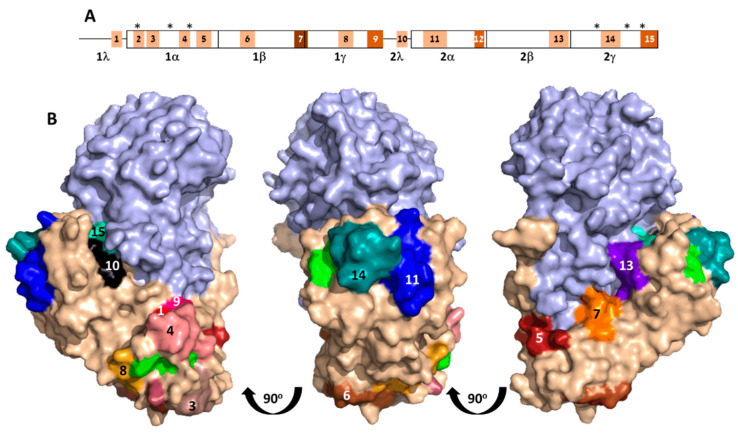
Immunodominant epitopes on ATB. (**A**) Linear depiction of two ATB subunit homologous globular domains and amino acid residues comprising galactose-binding pockets of each domain (marked by asterisks). Location of immunodominant epitopes (1–15) marked as shaded boxes, whereas shading tones represent number of sera that reacted with each epitope (pale to darkest for 1 to 4 sera, respectively). (**B**) Crystal structure of abrin (PDB 1abr; ATA in pale blue and ATB in light brown). Immunodominant epitopes color-coded and numbered; galactose-binding pockets in green.

ATB comprises two homologous globular domains [6], each containing a galactose-binding pocket. These domains can be further divided into four subdomains (Figure 4A), where a hydrophobic core is formed by Subdomains α, β, and γ, while Subdomain λ connects the two globular domains. Overall, mapped epitopes are distributed over the entire length of ATB, with Subdomain 1β being slightly more populated with interacting epitopes when compared to the 3 subdomains.

Visualization of the 15 epitopes on the crystal structure of abrin revealed that all but one are located on the surface of the toxin, securing their accessibility to antibody binding (Figure 4B). Epitope 12, however, is buried deep within the molecule, thus raising the question about its role as an antibody epitope. A possible explanation may rely on the fact that the sequence of this epitope (DGSI) also appears as a part of Epitope 13 (WVKFNDGSI) that is located at the surface of the toxin. It is thus possible that the antibodies that interacted with the peptides encompassing Epitope 12 were originally raised against Epitope 13.

As the main activity of ATB is to bind galactose moieties located on the cell surface and thus mediate toxin uptake, it was of interest to examine whether any of the identified ATB epitopes play a role in abrin neutralization by blocking its ability to bind galactose. ATB contains two potential galactose-binding sites, N51 and N260, for Subdomains 1 and 2, respectively [6]. In addition, on the basis of structure similarities to ricin, two residues (in each subdomain) were assumed to be involved in hydrogen bonding to the sugar (D27 and W42 for Domain 1; D239 and W253 for Domain 2; Figure 4). Indeed, the galactose-binding pocket of Domain 1 seemed to be populated by several of the ATB epitopes. First, sugar-binding Residue D27 is a part of Epitope 2, and Epitopes 4 and 8 surround the binding pocket. As for Domain 2, it appears that Epitope 14 is in close proximity to the second galactose-binding pocket, and can thus also be regarded as a putative neutralizing epitope. Although the main function of the ATB is to bind the cell membrane, it is highly possible that it also has a role in intracellular trafficking (mainly in the early endosomes) where it may interact with other proteins. Therefore, other epitopes, though located distally to the galactose-binding pockets, might also play a role in antibody-mediated abrin neutralization.

## 4. Conclusions

We characterized for the first time the polyclonal antibody response towards abrin, and identified the immunodominant epitopes of each of the toxin’s subunits. By screening with antibodies derived from two animal species (rabbits and mice) by different vaccination strategies, we increased the possibility to identify a wide coverage of epitopes. It is possible, however, that these antibody preparations also target nonlinear epitopes that cannot be identified by current method applied in this study. As abrin is considered an imminent biothreat agent, there is an ongoing effort to develop effective countermeasures to this toxin. Epitope mapping of the polyclonal antibodies in the sera of immunized animals enhances our knowledge regarding the antigenic moieties of abrin and provides important information for the development of such countermeasures. Indeed, two of the identified ATA immunodominant epitopes were previously shown to be the target of neutralizing monoclonal antibodies. In the future, the neutralization potency of the novel epitopes identified in this work (especially on ATB) will be evaluated. To conclude, the findings of this study provide useful information as part of an overall strategy to design improved vaccines and countermeasures to abrin.

## Figures and Tables

**Table 1:** Characteristics of antiabrin antibodies.

Serum	Binding (DIL_50_) ^a^	Neutralization (ED_50_) ^b^	B:N
R1	110,000	22,600	4.9
R2	27,000	8800	3.1
R3	153,400	112,300	1.4
M1	9000	960	9.4

^a^ Half-dilution values of sera in ELISA towards abrin; ^b^ serum dilutions that neutralize 50% of abrin activity in vitro.

**Table 2:** ATA immunodominant epitopes.

Epitope No.	Epitope Sequence	ATA Residue Number
1	EDRPI	1–5
2	KQFIEALR	18–25
3	IPVLP	36–40
4	TNAYV	71–75
5	GTQSY	81–85
6	DYLFTGT	96–102
7	GLQALT	130–135
8	QPDAAMISLE	186–195
9	QESVQD	206–211
10	PVIVD	226–230
11	CNPPN	247–251

**Table 3:** ATB immunodominant epitopes.

Epitope No.	Epitope Sequence	ATB Residue Number
1	VRIGG	16–20
2	VDVYD	26–30
3	NGYHNG	31–36
4	DRLEE	46–50
5	WTLKSDK	54–60
6	YAPGSYV	74–80
7	IWDNGT	97–102
8	MGGTLTV	119–125
9	QGWRTGN	134–140
10	VTSIS	146–150
11	QAQGSNVWMAD	158–168
12	DGSI	183–186
13	WVKFNDGSI	221–229
14	KGSDPSLKQ	241–249
15	QIWLTLF	261–267

## Supplementary Materials

The following are available online at https://www.mdpi.com/2073-4468/9/2/11/s1. Figure S1: Sequences of 15-mer peptides of ATA and ATB used for binding screening assay.
