# Supplementary material for: Mapping Immunodominant Antibody Epitopes of Abrin
Source: Antibodies (Basel). 2020 Apr 27;9(2):11. doi: 10.3390/antib9020011 (PMC7344891; doi:10.3390/antib9020011)
Supplement: Supplementary file 1 [file antibodies-09-00011-s001.pdf]

# Mapping the Immunodominant Antibody Epitopes of Abrin

**Ron Alcalay[1], Reut Falach[1], Yoav Gal[1], Anita Sapoznikov[1], Tamar Sabo[1], Chanoch Kronman[1] and Ohad Mazor[2,*]**

**Figure S1: Sequences of 15-mer peptides of ATA and ATB that were used for binding screening assay**

| Peptide No. | ATA |
|---|---|
| 1 | EDRPIKFSTEGATSQ |
| 2 | KFSTEGATSQSYKQF |
| 3 | GATSQSYKQFIEALR |
| 4 | SYKQFIEALRERLRG |
| 5 | IEALRERLRGGLIHD |
| 6 | ERLRGGLIHDIPVLP |
| 7 | GLIHDIPVLPDPTTL |
| 8 | IPVLPDPTTLQERNR |
| 9 | DPTTLQERNRYITVE |
| 10 | QERNRYITVELSNSD |
| 11 | YITVELSNSDTESIE |
| 12 | LSNSDTESIEVGIDV |
| 13 | TESIEVGIDVTNAYV |
| 14 | VGIDVTNAYVVAYRA |
| 15 | TNAYVVAYRAGTQSY |
| 16 | VAYRAGTQSYFLRDA |
| 17 | GTQSYFLRDAPSSAS |
| 18 | FLRDAPSSASDYLFT |
| 19 | PSSASDYLFTGTDQH |
| 20 | DYLFTGTDQHSLPFY |
| 21 | GTDQHSLPFYGTYGD |
| 22 | SLPFYGTYGDLERWA |
| 23 | GTYGDLERWAHQSRQ |
| 24 | LERWAHQSRQQIPLG |
| 25 | HQSRQQIPLGLQALT |
| 26 | QIPLGLQALTHGISF |
| 27 | LQALTHGISFFRSGG |
| 28 | HGISFFRSGGNDNEE |
| 29 | FRSGGNDNEEKARTL |
| 30 | NDNEEKARTLIVIIQ |
| 31 | KARTLIVIIQMVAEA |
| 32 | IVIIQMVAEAARFRY |
| 33 | MVAEAARFRYISNRV |
| 34 | ARFRYISNRVRVSIQ |
| 35 | ISNRVRVSIQTGTAF |
| 36 | RVSIQTGTAFQPDAA |
| 37 | TGTAFQPDAAMISLE |
| 38 | QPDAAMISLENNWDN |
| 39 | MISLENNWDNLSRGV |
| 40 | NNWDNLSRGVQESVQ |
| 41 | LSRGVQESVQDTFPN |
| 42 | QESVQDTFPNQVTLT |
| 43 | DTFPNQVTLTNIRNE |
| 44 | QVTLTNIRNEPVIVD |
| 45 | NIRNEPVIVDSLSHP |
| 46 | PVIVDSLSHPTVAVL |
| 47 | SLSHPTVAVLALMLF |
| 48 | TVAVLALMLFVCNPP |
| 49 | VAVLALMLFVCNPPN |

| Peptide No. | ATB |
|---|---|
| 1 | IVEKSKICSSRYEPT |
| 2 | KICSSRYEPTVRIGG |
| 3 | RYEPTVRIGGRDGMC |
| 4 | VRIGGRDGMCVDVYD |
| 5 | RDGMCVDVYDNGYHN |
| 6 | VDVYDNGYHNGNRII |
| 7 | NGYHNGNRIIMWKCK |
| 8 | GNRIIMWKCKDRLEE |
| 9 | MWKCKDRLEENQLWT |
| 10 | DRLEENQLWTLKSDK |
| 11 | NQLWTLKSDKTIRSN |
| 12 | LKSDKTIRSNGKCLT |
| 13 | TIRSNGKCLTTYGYA |
| 14 | GKCLTTYGYAPGSYV |
| 15 | TYGYAPGSYVMIYDC |
| 16 | PGSYVMIYDCTSAVA |
| 17 | MIYDCTSAVAEATYW |
| 18 | TSAVAEATYWEIWDN |
| 19 | EATYWEIWDNGTIIN |
| 20 | EIWDNGTIINPKSAL |
| 21 | GTIINPKSALVLSAE |
| 22 | PKSALVLSAESSSMG |
| 23 | VLSAESSSMGGTLTV |
| 24 | SSSMGGTLTVQTNEY |
| 25 | GTLTVQTNEYLMRQG |
| 26 | QTNEYLMRQGWRTGN |
| 27 | LMRQGWRTGNNTSPF |
| 28 | WRTGNNTSPFVTSIS |
| 29 | NTSPFVTSISGYSDL |
| 30 | VTSISGYSDLCMQAQ |
| 31 | GYSDLCMQAQGSNVW |
| 32 | CMQAQGSNVWMADCD |
| 33 | GSNVWMADCDSNKKE |
| 34 | MADCDSNKKEQQWAL |
| 35 | SNKKEQQWALYTDGS |
| 36 | QQWALYTDGSIRSVQ |
| 37 | YTDGSIRSVQNTNNC |
| 38 | IRSVQNTNNCLTSKD |
| 39 | NTNNCLTSKDHKQGS |
| 40 | LTSKDHKQGSTILLM |
| 41 | HKQGSTILLMGCSNG |
| 42 | TILLMGCSNGWASQR |
| 43 | GCSNGWASQRWVFKN |
| 44 | WASQRWVFKNDGSIY |
| 45 | WVFKNDGSIYSLYDD |
| 46 | DGSIYSLYDDMVMDV |
| 47 | SLYDDMVMDVKGSDP |
| 48 | MVMDVKGSDPSLKQI |
| 49 | KGSDPSLKQIILWPY |
| 50 | SLKQIILWPYTGKPN |
| 51 | ILWPYTGKPNQIWLT |
| 52 | WPYTGKPNQIWLTLF |
